# Rapid evolution of a novel protective symbiont into keystone taxon in *Caenorhabditis elegans* microbiota

**DOI:** 10.1038/s41598-022-18269-7

**Published:** 2022-08-18

**Authors:** Alejandra Wu-Chuang, Kieran A. Bates, Dasiel Obregon, Agustín Estrada-Peña, Kayla C. King, Alejandro Cabezas-Cruz

**Affiliations:** 1 Anses, INRAE, Ecole Nationale Vétérinaire d’Alfort, UMR BIPAR, Laboratoire de Santé Animale, 94700 Maisons-Alfort, France; 2 Department of Zoology, University of Oxford, Oxford, OX1 3SZ UK; 3 School of Environmental Sciences, University of Guelph, Guelph, ON Canada; 4 Faculty of Veterinary Medicine, University of Zaragoza, Zaragoza, Spain

**Keywords:** Coevolution, Experimental evolution, Bacteria, Microbial communities, Environmental microbiology, Pathogens, Ecology

## Abstract

Protective microbes have a major role in shaping host–pathogen interactions, but their relative importance in the structure of the host microbiota remains unclear. Here, we used a network approach to characterize the impact of a novel, experimentally evolved ‘protective microbial symbiont’ (*Enterococcus faecalis*) on the structure and predicted function of the natural microbiota of the model organism *Caenorhabditis elegans*. We used microbial network analysis to identify keystone taxa and describe the hierarchical placement of protective and non-protective symbionts in the microbiota. We found that early colonization with symbionts produce statistically significant changes in the structure of the community. Notably, only the protective *E. faecalis* became a keystone taxon in the nematode microbiota. Non-protective lineages of the same bacterial species remained comparatively unimportant to the community. Prediction of functional profiles in bacterial communities using PICRUSt2 showed that the presence of highly protective *E. faecalis* decreased the abundance of ergothioneine (EGT) biosynthesis pathway involved in the synthesis of the antioxidant molecule EGT, a potential public good. These data show that in addition to direct antagonism with virulent pathogens, keystone protective symbionts are linked to modified bacterial community structure and possible reductions in public goods, potentially driving decreased antioxidant defense. We suggest that this response could suppress infection via wholesale microbial community changes to further benefit the host. These findings extend the concept of protective symbionts beyond bodyguards to ecosystem engineers.

## Introduction

Animals have complex ecological interactions with their associated microbes^1^. Some of these micro-organisms are known as protective symbionts as they can defend their hosts against pathogen infection^2,3^. Whether protective symbionts were acquired by the host from the environment or inherited^3^, interactions with other colonizing microbes may occur within the host. The protective phenotype may emerge from host-microbe interactions^4^. Alternatively, direct interactions between the symbionts and pathogens can result in disease suppression via resource or interference competition^5,6^.

Whilst some protective symbionts are intracellular^7^, others may be embedded and interact with other host-microbiota components^8,9^. It has been previously shown that the protective effect can be maintained despite multi-species interactions within the microbiota, with the symbionts having minimal impact on native microbial diversity^10^. Whether protective symbionts can evolve to become important factors in the microbial community hierarchy is unclear. Some keystone taxa, *with a major influence on microbiome structure and function at a particular space or time*^11^, have been linked with host defense^12^. For example, keystone taxa are associated with protection against ‘bacterial wilt’, a common soil-borne disease caused by *Ralstonia solanacearum* in tobacco plants^12^. Other keystone species in the microbiota play key roles in the regulation of human gut metabolism^13^. The manipulation of keystone taxa is a promising approach to induce changes in the microbial community composition and functioning via taxa depletion or inoculation. Depletion of the keystone genera *Escherichia*-*Shigella* via host antibodies produced major shifts in the tick microbiota and affected tick feeding^14,15^. Moreover, inoculation of three *Pseudomonas* isolates, *P. lurida* FGD5-2, *P. koreensis* HCH2-3, and *P. rhodesiae* MTD4-1 displayed strong inhibitory effects on *R. solanacearum*^12^. Keystone taxa provide important services to the microbial community but can also participate in host defense.

The model organism *Caenorhabditis elegans* has a natural, resident microbiota^1,16,17^. A meta-analysis of *C. elegans* microbiota data shows that, regardless of the origin of the worms, whether field or laboratory isolates, *C. elegans* exhibit a core microbiota dominated by the bacterial families as Enterobacteriaceae, Pseudomonaceae, Xanthomonodaceae, Sphingobacteriaceae, Weeksellaceae and Flavobacteriaceae^18^. However, rare taxa, such as Acetobacteriaceae, were also consistently recovered from the *C. elegans* microbiota^18^. Acetobacteriaceae was identified as a possible keystone taxon associated with large populations of *C. elegans* proliferating in rotting apples^1^, which suggests a beneficial role of this keystone taxon on host physiology. Gut microbiota also contributes to *C. elegans* immunity and resistance to bacterial infections. King et al.^19^ demonstrated that co-adaptation between *C. elegans* and a novel, resident bacterium. *Enterococcus faecalis,* during infection by the pathogen *Staphylococcus aureus* can select for host-protection by *E. faecalis.*^19^. When *C. elegans* is colonised by evolved, protective *E. faecalis,* and subsequently exposed to a complex compost microbiota, there is minimal impact on the host microbiota diversity^10^.

Inference of microbial co-occurrence networks is a powerful tool for the study of microbial community structure and its dynamics^20,21^. Based on abundance data, obtained from high-throughput sequencing technologies, significant patterns of co-occurrence between microbial taxa can be detected and illustrated as networks^20^. Thus, nodes and links in a microbial network represent bacterial taxa and their respective co-occurrences^22^. Besides the prediction of bacterial associations, microbial co-occurrence network analysis offers the possibility to statistically identify keystones taxa. Although there is not a consensus on the metrics used for their identification, it is clear that keystone taxa are highly influential bacteria on the microbial community structure and functioning^11,21,23^. Apart from deciphering microbial community assembly patterns, 16S rRNA data can also be used for the prediction of metabolic function by matching taxonomic data to metabolic reference databases^24,25^. Thus, the exploration of microbial co-occurrence networks, identification of keystone taxa and functional prediction can give a comprehensive insight into the dynamics of the microbiota when a protective symbiont is introduced.

Here, we used a network approach to analyse 16S rRNA metabarcoding data from Dahan et al.^10^ and further characterized the impact of experimentally adapted, protective microbial lineages in the subsequent structure of the *C. elegans-*associated microbial community. Although broad diversity metrics (i.e., alpha/beta diversity) were unchanged^10^, we hypothesized that the introduction of highly protective lineages could alter microbe-microbe or microbe-host interactions and yield differences in overall community function that cannot be detected by taxonomy alone. We found that newly introduced microbes with strong protective phenotypes can become keystone taxa. Our results suggest that these symbionts play important roles in microbial community structure, as well as in host health. Within the text, “microbiome” refers to the microorganisms and their metagenome whereas “microbiota” refers only to the microbes themselves.

## Methods

### Original 16S rRNA datasets

**Figure 1:**
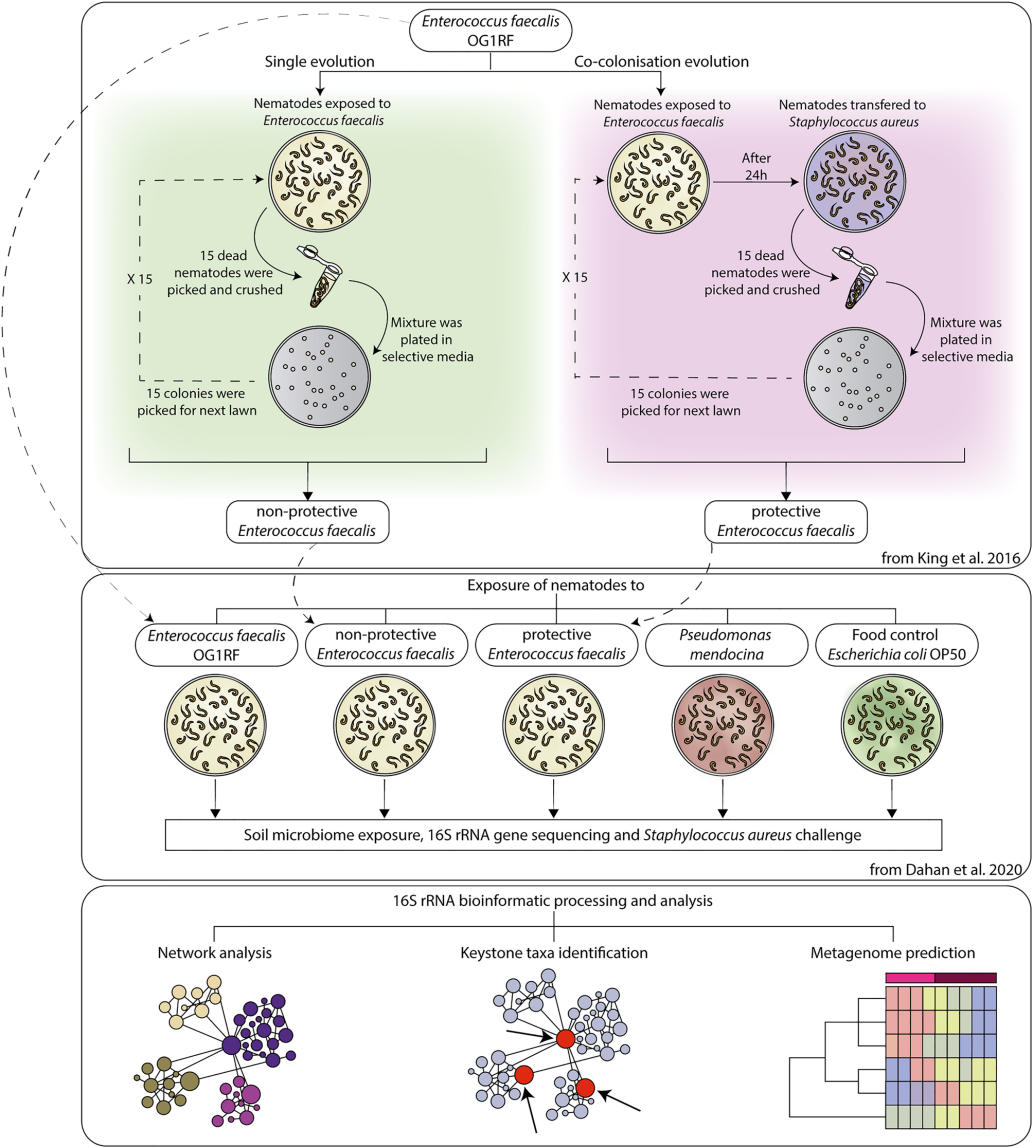
Graphical summary of the origin of different symbionts exposed to *C. elegans*. (Top panel) *E. faecalis* derived from a single ancestor clone, *E. faecalis* OG1RF, was used for an experimental evolution in *C. elegans* over 15 generations. In the experiment done by King et al.^19^, *C. elegans* was exposed to *E. faecalis* in the absence (green panel) or presence (pink panel) of a fixed, non-evolving pathogen *S. aureus*. Then, bacteria-killed nematodes were picked and crushed to release the pathogens from inside the carcass and the suspension was streaked on selective media to isolate populations of *E. faecalis*. Subsequently, 15 colonies were selected to make a lawn for the next exposure generation. From the single evolution, a non-protective *E. faecalis* was obtained while from the co-colonisation evolution, the protective *E. faecalis* strain was obtained. (Middle panel) The different strains of *E. faecalis* were then used for the exposition to *C. elegans,* in an independent experiment done by Dahan et al.^10^, alongside with *P. mendocina*, known to be protective against *P. aeruginosa*. *E. coli* OP50 was used as food control. (Bottom panel) 16s rRNA gene sequencing from *C. elegans* exposed to different symbionts were used for network analysis, keystone taxa identification and metagenomic prediction in the present study.

In the present study, we used available 16S rRNA amplicon sequence datasets^10^. In the original study, Dahan et al.^10^ tested the impact of different bacterial strains on the microbiota of *C. elegans.* To this aim, *E. faecalis* OG1RF, originally isolated from the human gastrointestinal tract, was used as the ‘ancestral strain’ from which two lineages were derived by serial passages (non-protective and protective, Fig. 1A). The ‘non-protective *E. faecalis*’ evolved from ancestral *E. faecalis* in the absence of pathogen coinfection and did not confer protection to *C. elegans*. The ‘protective *E. faecalis*’ evolved from ancestral *E. faecalis* in the presence of a pathogenic *Staphylococcus aureaus* strain, and conferred protection against pathogen infection^19^ (Fig. 1A).

In addition to using the ancestral *E. faecalis*, non-protective *E. faecalis* and protective *E. faecalis*, Dahan et al.^10^ tested the impact of *Pseudomonas mendocina* of the microbiota of *C. elegans* (Fig. 1B). *Pseudomonas mendocina* was selected based on its capability to elicit host defenses against the pathogen *Pseudomonas aeruginosa* and was originally isolated from soil^26^. *Escherichia coli* OP50 was used as a control since these bacteria are used to feed the nematodes (Fig. 1B). The strains were selected in Dahan et al.^10^ based on having extreme differences in protective phenotype amongst the biological replicates in King et al.^19^. These phenotypic differences are well-defined and supported by genomic and biochemical analyses, as in King et al.^19^. Moreover, multiple replicates of each strain treatment were conducted in Dahan et al.^10^. In Dahan et al.^10^, *C. elegans* was colonized with the three different strains of *E. faecalis* or *P. mendocina*. Then, the worms were exposed to a complex compost microbiota and subsequent 16S rRNA sequencing was carried out (Fig. 1B). Datasets were generated by amplification of the 16S rRNA hypervariable region four (V4) using the barcoded universal primers 515F/806R and sequenced in an Illumina MiSeq system^10^. The raw sequence data are available in the EMBL-EBI repository under the project accession number PRJEB26987.

### Analysis of 16S rRNA amplicon sequences

For this study, the 16S rRNA sequences were downloaded from EMBL-EBI repository. Using DADA2 software^27^ implemented in QIIME2 version 2019.7^28^, 16S rRNA sequences were first demultiplexed and then quality trimmed based on the average quality per base of the forward and reverse reads. The first 23 nucleotides were truncated, and the total length was trimmed at 220 and 205 in forward and reverse reads, respectively. Consequently, reads were merged and chimeric variants were removed. The resulting representative sequences were taxonomically assigned using a pre-trained naïve Bayes taxonomic classifier^29^ based on SILVA database version 132^30^ and the 515F/806R primer set. The resulting taxonomic data tables were collapsed at genus level and taxa with less than 10 total reads and presents in less than 30% of samples of each dataset were removed. The taxonomic data tables were used for network analysis, keystone taxa identification and metagenome prediction (Fig. 1C).

### Construction of bacterial co-occurrence networks, identification of keystone taxa and attack tolerance test

Co-occurrence network analyses were performed using the Sparse Correlations for Compositional data (SparCC) method^31^ implemented in R studio^32^. Taxonomic data tables were used to calculate the correlation matrix. Correlation coefficients with magnitude > 0.5 or < − 0.5 were selected. Network visualization and calculation of topological features and taxa connectedness (i.e., number of nodes and edges, modularity, network diameter, average degree, weighted degree, clustering coefficient and centrality metrics) was performed using the software Gephi 0.9.2^33^. To infer keystone taxa, we used the combination of three parameters as previously described^14,15^: (i) eigencentrality (index that indicates the influence of the node on the co-occurrence network) values equal or higher than 0.75, (ii) clr-based abundance higher than the mean clr value (i.e., 6), and (iii) ubiquitousness, (i.e., presence of the bacteria in 100% of the samples of a given experimental condition). Scatter plots were done using the software GraphPad 8 Prism (GraphPad Software Inc., San Diego, CA, USA). The robustness of co-occurrence networks was tested with an attack tolerance test using the package NetSwan for R^34^. For this, networks were subjected to systematic removal of nodes using three different scenarios: (i) a random attack with 100 iterations, (ii) a directed attack where nodes are removed in decreasing order of their betweenness centrality (BNC) value (i.e., number of times a node is found on the shortest path between other nodes) and (iii) a cascading attack where BNC value are recalculated after each node removed.

### Differential network analysis

Comparison of the similarity of the most central nodes between two networks was done with the package “NetCoMi”^35^ in R studio using the read count taxonomic tables. “Most central” nodes are defined as those nodes with a centrality value above the empirical 75% quartile. The comparison returns Jaccard's indexes for each of four local measures (i.e., degree, betweenness centrality, closeness centrality, eigenvector centrality) of the sets of most central nodes as well as for the sets of hub taxa between the two networks compared. Thus, the Jaccard’s index express the similarity of the sets of most central nodes as well as the sets of hub taxa between the two networks. Jaccard index of 0 indicates completely different sets while a value of 1 indicates equal sets of most central nodes or hub taxa between the compared networks^35^.

### Prediction of functional traits in *C. elegans* microbiome

PICRUSt2^24^ was used to predict the functional profile of bacterial communities based on 16S rRNA gene sequences. Briefly, amplicon sequence variants (ASVs) were placed into a reference tree (NSTI cut-off value of 2), which was then used to infer gene family copy numbers for each ASV. The Kyoto Encyclopedia of Genes and Genomes (KEGG) orthologs (KO)^36^, Enzyme Classification numbers (EC) and Cluster of Orthologous Genes (COGs)^37^ were used for the functional annotations of enzymes and pathways. With enzyme information, pathway profiles were inferred using structured pathway mapping based on the MetaCyc database^38^. Metacyc pathway was calculated based on the predicted EC number abundances.

### Statistical analysis

Taxonomic, pathway and enzyme data tables, which consisted of sequencing-read counts or inferred gene families and functional gene abundance respectively were used as input of the R package ‘ALDEx2’^39^ for differential abundance comparisons. To avoid bias in the differential analyses due to compositional nature of the sequence data, ALDEx2 method performs centered log-ratio (clr) transformation of feature counts in each sample^40^. A comparison of bacterial genera, pathways or enzyme clr values was done among all the experimental groups, using Kruskal–Wallis test for multiple comparisons. For further analyses, we performed pairwise comparison between groups (i.e., protective *E. faecalis* vs. non-protective *E. faecalis* groups), which was performed using Welch's t-test. Heatmaps were built from euclidean distances of clr values to represent pathway and enzyme abundance using the R package ‘Heatplus’^41^.

The number of shared keystone taxa in all experimental groups, shared direct neighbor of the reference taxon *Enterococcus* in the three *E. faecalis*-exposed groups and shared differential pathways of pairwise comparisons between the three different strains of *E. faecalis* or *P. mendocina* compared to the control group was visualized using Venn diagrams implemented in the online tool http://bioinformatics.psb.ugent.be/webtools/Venn/.

Alpha and beta-diversity of functional genes were carried out on rarified EC (Enzyme commission) tables. The alpha-diversity was explored using Shannon’s diversity index^42^, Pielou’s evenness^43^ and richness^44^ metrics. Differences in alpha-diversity metrics between groups were tested using a Kruskal–Wallis method. Subsequently, Kruskal–Wallis pairwise comparisons between groups hypothesized to be different (i.e., protective vs. non-protective *E. faecalis*) was performed which served as a post-hoc test in this contest. Beta-diversity was explored using the Bray–Curtis dissimilarity and compared among all the groups using a PERMANOVA test. Betadisper function in R was used for the construction of PCoA plot based on Bray–Curtis distance matrix and an analysis of variance (ANOVA) test was used for comparison of the dispersion of the samples between the groups. For differential pathway and enzyme abundance comparison, a Kruskal–Wallis one-way ANOVA was used. In addition, a Welch’s t-test was used to determine significant pathway abundance differences in pairwise comparisons. For testing similarity of most central nodes, two p-values P(J ≤ j) and P(J ≥ j) for each Jaccard’s index, which represent the probability that the observed value of Jaccard’s index is “less than or equal” or “higher than or equal”, respectively, to the Jaccard value expected at random, were calculated. Differences were considered significant when p-value < 0.05.

## Results

### Impact of early colonization with symbionts on the structure of *C. elegans* microbiota

**Figure 2:**
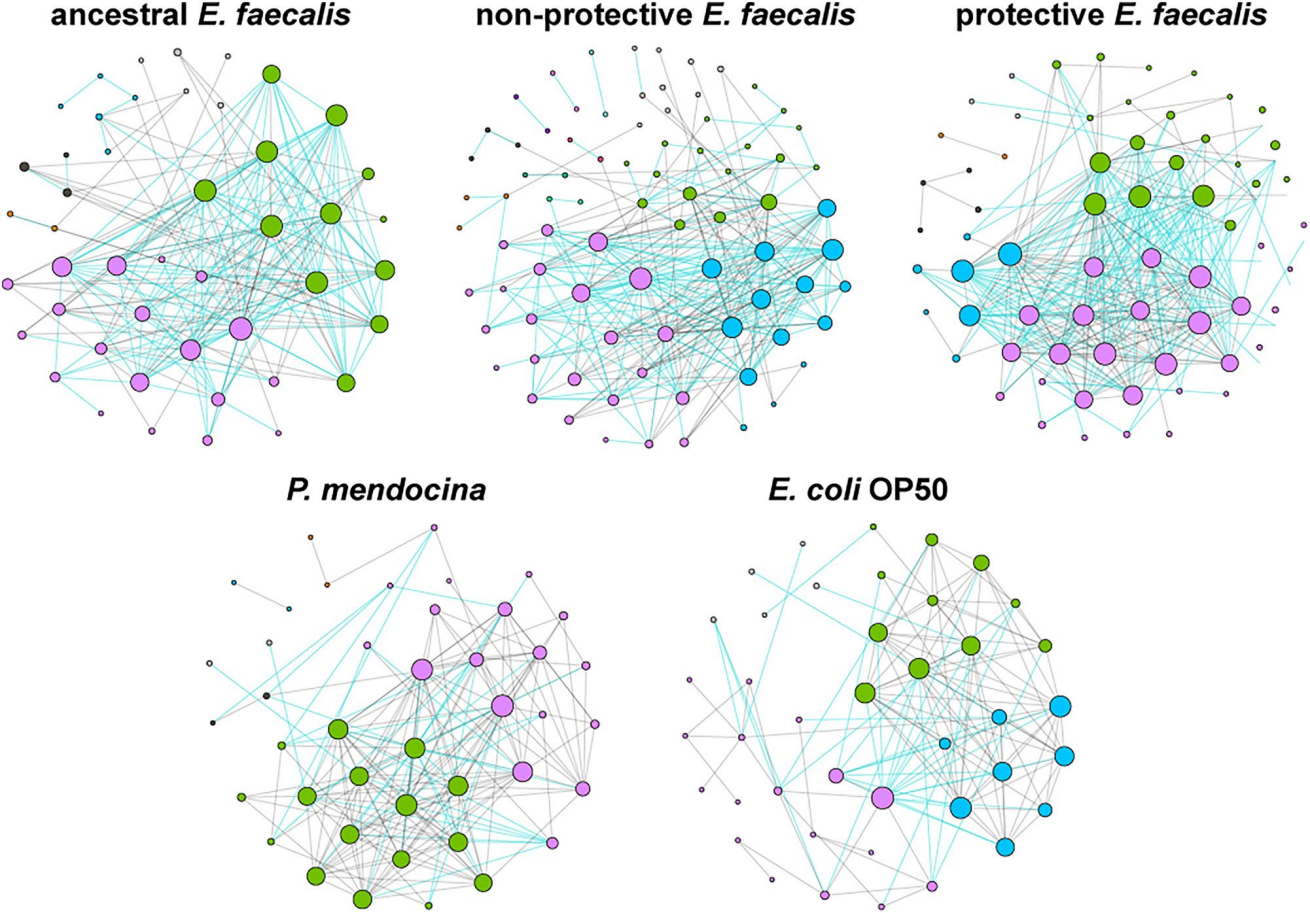
Microbial co-occurrence networks of *C. elegans* exposed to different bacteria. Bacterial co-occurrence networks based on 16S sequences obtained from C. elegans exposed to different populations of *E. faecalis* (non-protective *E. faecalis*, protective *E. faecalis* and ancestral *E. faecalis*), *P. mendocina* and *E. coli* OP50. Nodes represent bacterial taxa and edges stand for co-occurrence correlation (SparCC > 0.5 or < − 0.5). Node size is proportional to the eigenvector centrality and node color is based on the modularity class. Thus, nodes with the same color belong to the same cluster. Gray and green edges are connecting links with negative and positive interactions, respectively. Only nodes with at least one connecting edge are displayed.

Ecological interactions of early bacterial colonizers with *C. elegans* microbiota were inferred by co-occurrence networks. Visual inspection of the networks showed distinct patterns of bacterial taxa co-occurrence in the different experimental groups (Fig. 2). The topological features of ancestral *E. faecalis*, non-protective *E. faecalis*, protective *E. faecalis* and *P. mendocina* networks reveals an increased number of edges and nodes compared with the control network, *E. coli* OP50 (Table 1). Jaccard index was used to test for similarities of the most central nodes in the networks. Except for the Jaccard index of betweenness centrality, the observed Jaccard index for all the other local measures were significantly higher than expected by random in the ancestral *E. faecalis*, non-protective *E. faecalis*, protective *E. faecalis* and *P. mendocina* networks compared to the control group (Supplementary Table S1). Jaccard index revealed statistical differences in several network parameters between the symbiotic and control groups which further confirms our observations of the microbial co-occurrence networks. These results suggest that the early colonization with symbionts modifies the structure of *C. elegans* microbial communities compared to the control communities.

**Table 1:** Topological features of the microbial co-occurrence networks.

Topological features	Experimental groups				
	Ancestral *E. faecalis*	Non-protective *E. faecalis*	Protective *E. faecalis*	*P. mendocina*	*E. coli* OP50
Nodes^a^;	190	191	207	183	131
Edges^b^	197	288	315	197	135
Positives	123 (62%)	175 (61%)	171 (54%)	154 (78%)	98 (73%)
Negatives	74 (38%)	113 (39%)	144 (46%)	43 (22%)	37 (27%)
Modularity^c^	0.493	1.057	0.979	0.362	0.612
Modules^d^	11	19	18	7	8
Network diameter^e^	7	7	7	6	7
Average degree^f^	2.074	3.016	3.043	2.153	2.061
Weighted degree^g^	0.43	0.446	0.256	0.778	0.616
Clustering coefficient^h^	0.644	0.488	0.626	0.692	0.475

^a^Nodes represents bacterial taxa with co-ocurrence correlation SparCC > or < − 0.5.

^b^Edges represent the number of connections/correlations.

^c^Modularity is the strength of division of a network into modules.

^d^Modules are sub-communities of bacteria that co-occur more frequently among each other than with other taxa.

^e^Network diameter is the shortest path between the two most separated nodes.

^f^Average degree is the average number of links per node.

^g^Weighted degree is the sum of the weight of all the edges connected to a node.

^h^Clustering coefficient is the degree to which nodes in a network tend to form clusters.

We next focused on the comparisons between microbiomes in the two evolved symbiont treatments and the ancestral treatment to study the possible impact of evolutionary history on microbe-microbe interactions. Jaccard index comparisons showed significant differences between evolved and ancestral *E. faecalis* networks for all the local measures, except the betweenness centrality. Only in the protective compared to the non-protective *E. faecalis* network, the Jaccard index for betweenness centrality was significantly higher than expected by random (Supplementary Table S1). Additionally, the network of the protective *E. faecalis* had the highest number of edges, nodes and average degree, while the highest number of modules was observed in the non-protective *E. faecalis* network (Table 1). This result suggests that early colonization with the evolved symbionts induced strain-specific modifications to the structure of *C. elegans* microbiota, compared to that induced by the ancestral strain.

**Figure 3:**
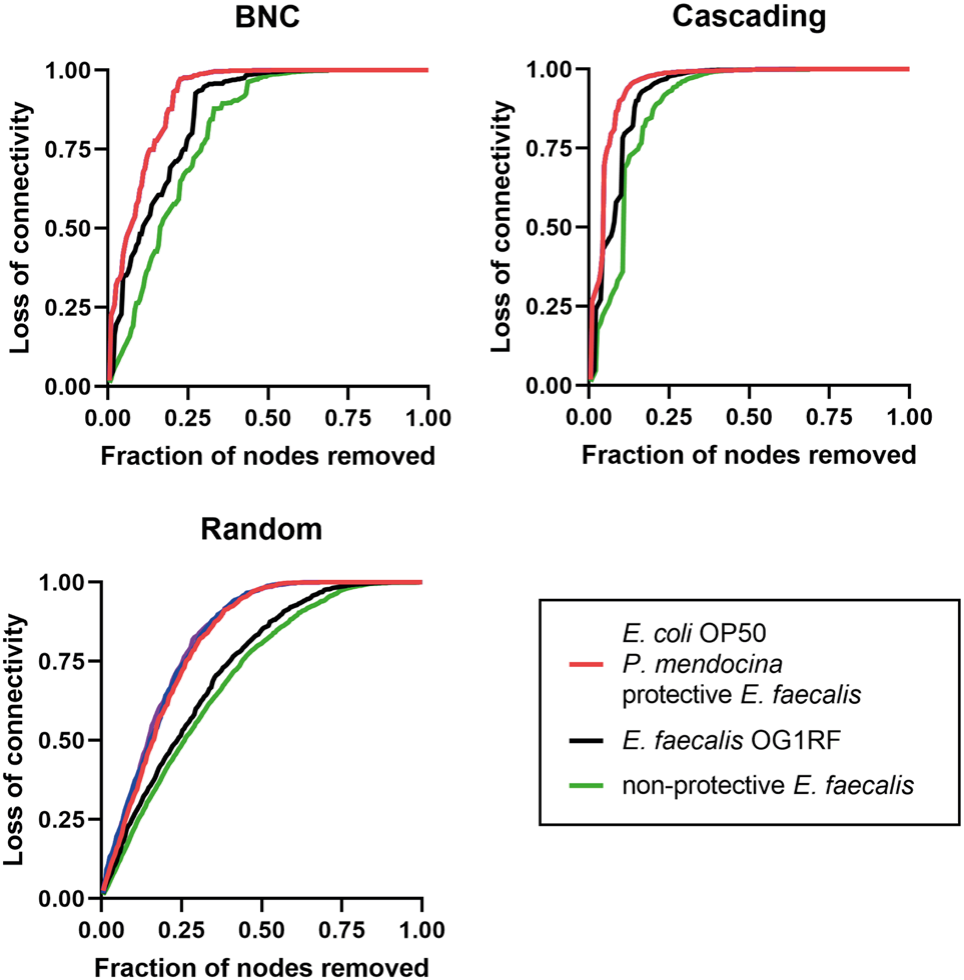
Comparison of network tolerance to taxa removal based on different types of attack. The resistance of the networks to directed (left), cascading (middle) or random (right), removal of nodes was measured in non-protective *E. faecalis* (green line), ancestral *E. faecalis* (black line), *E. coli* OP50*, P. mendocina,* and protective *E. faecalis* (red line) networks. Nodes removal was based on their betweenness centrality (BNC) value. Loss of connectivity values range between 0 (maximum of connectivity between nodes) and 1 (total disconnection between nodes).

The robustness of the networks was tested by measuring their tolerance to random or directed taxa removal. Networks of *C. elegans* exposed to different symbionts and subjected to random removal of taxa presented the highest tolerance, where a loss of ~ 85% of network connectivity was obtained after random removal of 50% of the nodes (Supplementary Fig. S1). Removal of taxa according to decreasing BNC produced a higher loss of connectivity in every network (Supplementary Fig. S1). However, the lowest tolerance was observed in all the networks induced by a cascading attack (Supplementary Fig. S1). Comparison of the loss of connectivity of the networks of all experimental groups showed that, independently of the type of attack, the *E. coli* OP50, *P. mendocina* and protective *E. faecalis* networks presented the lowest tolerances to taxa removal, followed by the ancestral *E. faecalis* network (Fig. 3). The non-protective *E. faecalis* network presented the highest tolerance to taxa removal (Fig. 3). These results suggest that the robustness of *C. elegans* networks is strain-specific and that the worm microbiota exposed to protective *E. faecalis* is as robust as the non-altered control microbiota.

### Impact of symbionts on keystone taxa and the hierarchical organization of *C. elegans* microbiota

**Figure 4:**
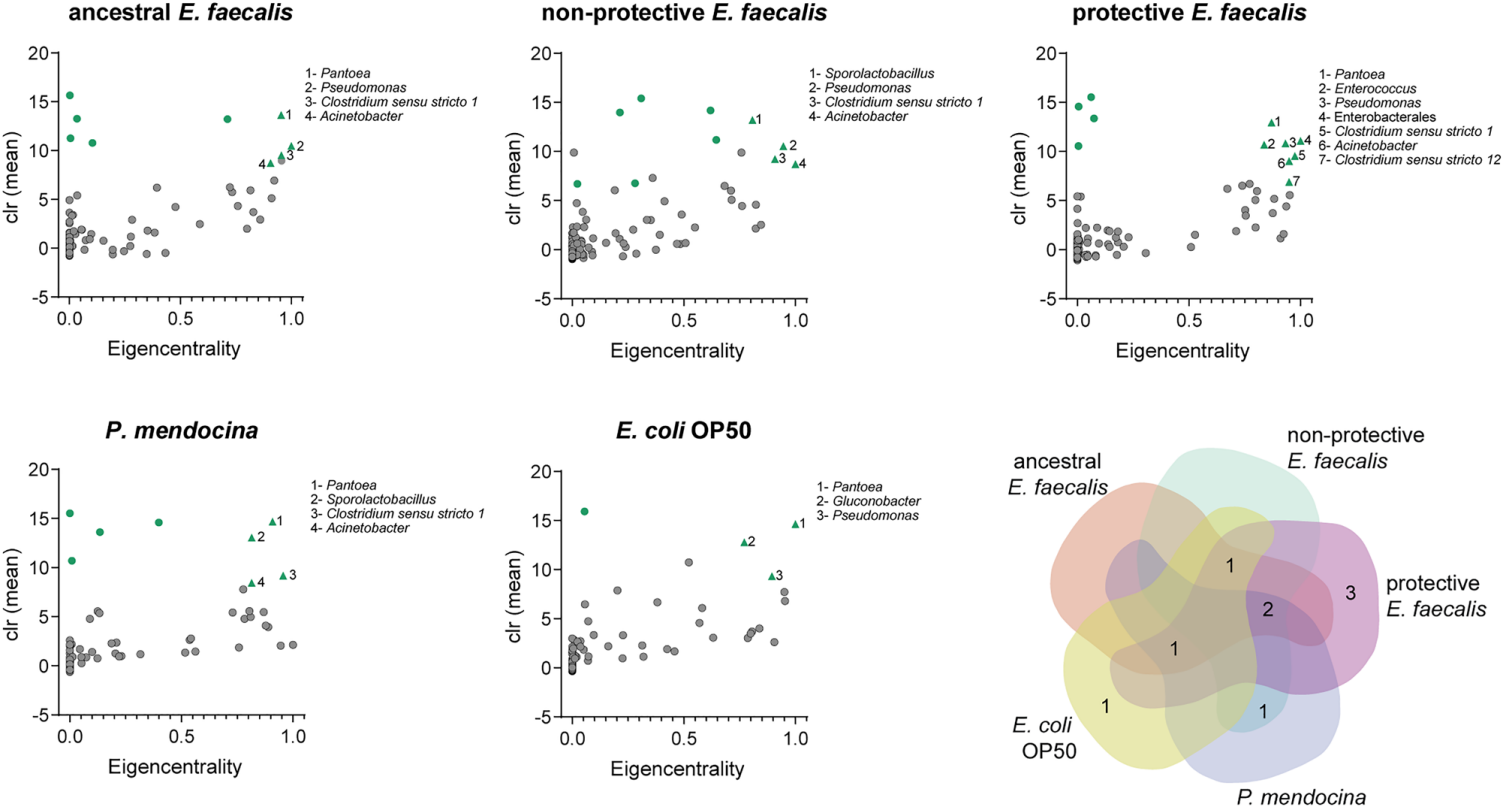
Identification of keystone taxa from microbial networks of *C. elegans* exposed to different bacteria. Scatter plot of the mean relative abundance (expressed as clr) vs. the eigenvector centrality of the bacterial taxa (dots or triangles) found in the microbial co-occurrence networks of *C. elegans* exposed to different symbionts (different *E. faecalis* populations or *P. mendocina*) and *E. coli* OP50. Green dots or triangles represent ubiquitous bacteria (i.e., taxa that were found across all the samples). Cutoff value of 6 were set for the mean relative abundance and 0.75 for the eigenvector centrality. Ubiquitous bacterial taxa with mean relative abundance and eigenvector centrality equal or higher than the cutoff values were considered as keystone taxa (Green triangles). List of keystone taxa is displayed next to the scatter plot. The Venn diagram shows the number of keystone taxa that are shared by or unique in the different experimental groups.

To define changes in the hierarchical organization of *C. elegans* microbiota, we sought to identify keystone taxa from microbial co-occurrence networks. Ubiquitous bacteria with high eigenvector centrality values (≥ 0.75) and relative abundance (clr > 6) in the network were considered as keystone taxa. We found three and seven keystone taxa in the networks of *C. elegans* exposed to *E. coli* OP50 and the protective *E. faecalis*, respectively (Fig. 4). The remaining networks (ancestral *E. faecalis*, non-protective *E. faecalis* and *P. mendocina*) contained four keystone taxa each (Fig. 4). A comparison of the different keystone taxa in all the experimental groups showed that the protective *E. faecalis* network presented 3 unique keystone taxa (i.e., *Clostridium *sensu stricto 12, *Enterococcus* and Enterobacterales); *E. coli* OP50 network also presented one unique keystone taxa (i.e., *Glunocobacter*) while the remaining networks did not harbor unique keystone taxa (Fig. 4). Interestingly, the reference taxon *Enterococcus* became a keystone only in the protective *E. faecalis* group, and not in the microbial networks of the ancestral *E. faecalis* or non-protective *E. faecalis* groups. It is noteworthy that *Pseudomonas* was found as a keystone taxon in all networks except in the *P. mendocina* group (Fig. 4).

For further analysis of the microbial community structure, we constructed sub-networks containing only the keystone taxa and their direct neighbors in all the experimental conditions. We referred to them as ‘keystone sub-networks’ (Supplementary Fig. S2). Visual inspection of the keystone sub-networks showed distinct patterns of bacterial taxa co-occurrence in the different experimental groups. Bacterial members and edges of these keystone sub-networks represented less than 20% of nodes and less than 50% of the edges of the full community in all the experimental groups. Furthermore, visual inspection of each keystone sub-network showed that all keystone taxa were connected to each other. These results suggest that symbiont colonization alter the hierarchical organization of *C. elegans* microbiota.

### Local connectivity of symbionts in *C. elegans* microbiota

**Figure 5:**
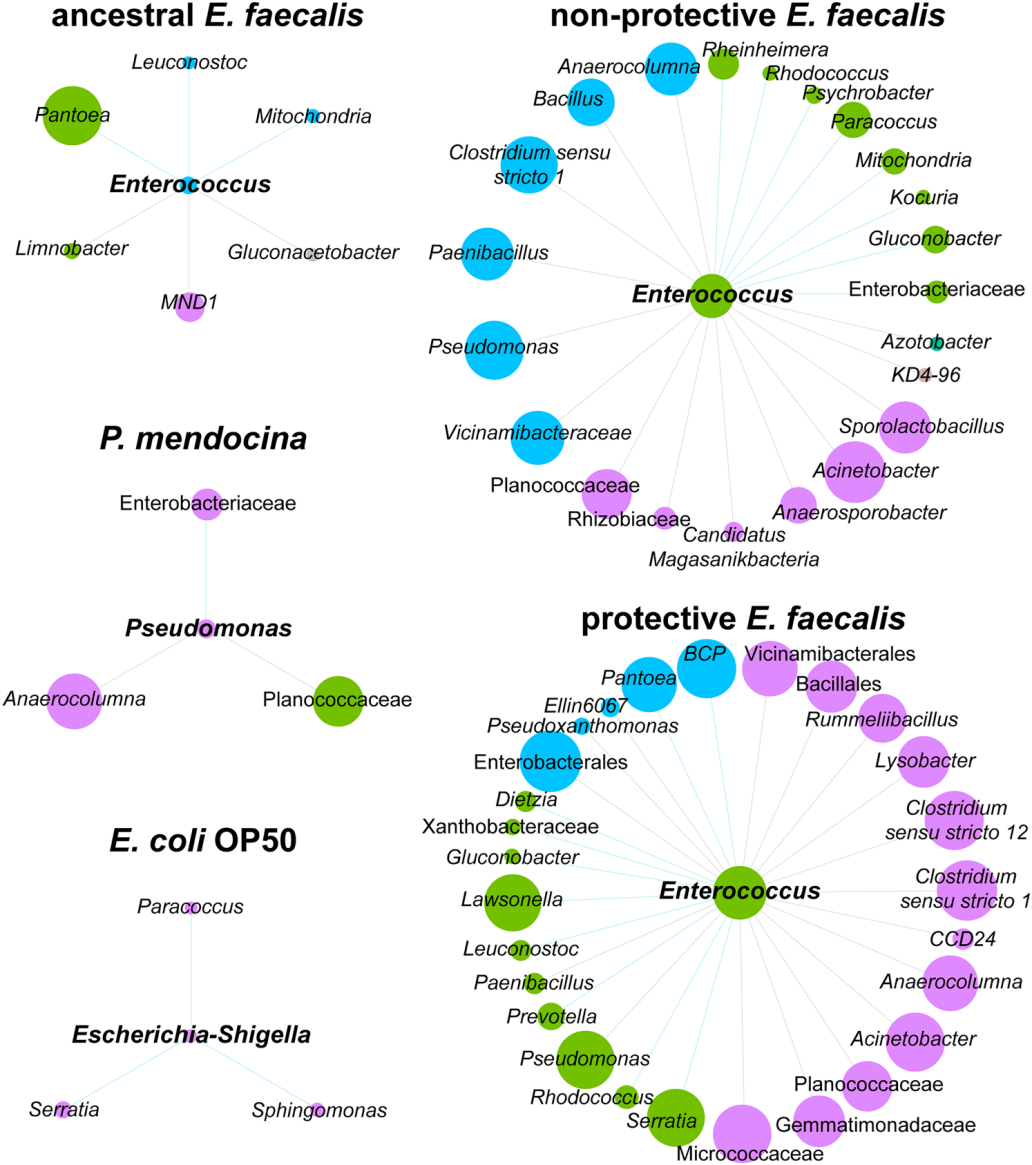
Sub-networks of the local connectivity of *Enterococcus, Pseudomonas* and *Escherichia-Shigella* in the co-occurrence networks. The direct neighbors of *Enterococcus*, *Pseudomonas* and *Escherichia-Shigella* were identified in the bacterial co-occurrence networks of *C. elegans* exposed to different strains of *E. faecalis, P. mendocina* as well as the food control *E. coli* OP50. Positive and negative interactions between co-occurring bacteria are represented by the green and gray edges, respectively. Node size is proportional to the eigenvector centrality and node color is based on the modularity class.

Reference taxa (i.e., *Enterococcus*, *Pseudomonas* and *Escherichia-Shigella*) were defined here as the ‘genera’ taxonomic classification of the symbionts in each experimental group. The impact of early symbiont colonization on the local connectivity of reference taxa was measured using sub-networks derived from the global networks and that represent the direct neighbors around these taxa (Fig. 5). Visual inspection showed differences in the sub-network structures, reflecting potential changes in local connectivity of symbionts. *Pseudomonas* and *Escherichia*-*Shigella* were directly connected to only three taxa each. *Enterococcus* had the higher numbers of direct neighbors in the sub-networks of non-protective (22 taxa) and protective (27 taxa) *E. faecalis*, compared to the ancestral *E. faecalis* (six taxa). The results suggest that evolved symbionts establish more microbe-microbe interactions with the bacterial microbiota of *C. elegans* than the ancestor.

**Figure 6:**
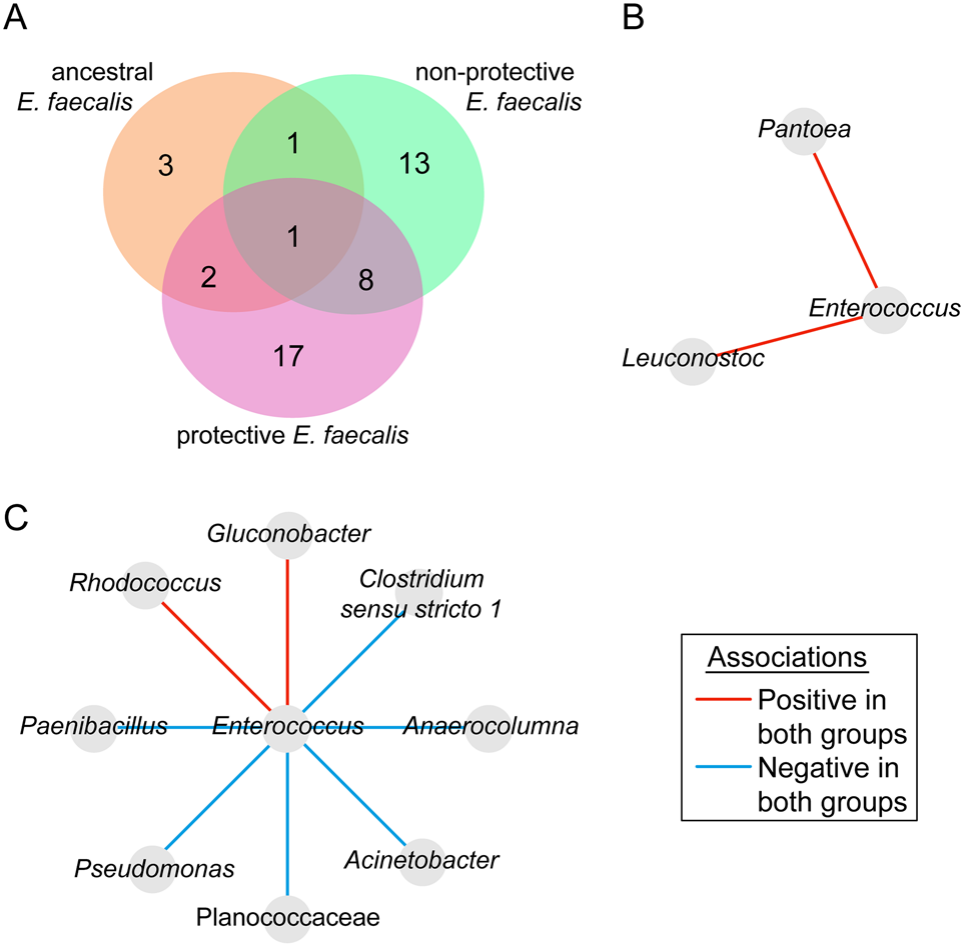
Number and associations patterns of shared neighbors of the taxon *Enterococcus*. Venn diagram showing the number of bacteria that are common or unique among the taxa directly connected to *Enterococcus* in the ancestral *E. faecalis*, non-protective *E. faecalis* and protective *E. faecalis* exposure groups (**A**). Direction of associations of common direct neighbor between the ancestral *vs*. protective *E. faecalis* groups (**B**) and non-protective *E. faecalis vs*. protective *E. faecalis* groups (**C**). *Mitochondria* stands for *C. elegans* mitochondria.

Further characterization of potential microbial interactions of *E. faecalis* was achieved by comparing the taxonomic identity of the direct neighbors of *Enterococcus* in the sub-networks of the three *E. faecalis* groups. The results revealed that the direct neighbors were mostly unique for each symbiont strain (Fig. 6A). A detailed pairwise comparison of the co-occurrence correlation between the reference taxon *Enterococcus* and the common taxa between the two compared groups: ancestral *E. faecalis* vs protective *E. faecalis* (Fig. 6B) and non-protective *E. faecalis* and protective *E. faecalis* (Fig. 6C) revealed that the type of connection was kept in the two comparisons. This result suggests that *Enterococcus* maintains common ecological associations with specific microbial members in the different groups. Changes in global (Fig. 2) and local (Fig. 5) network structure could be accompanied by hierarchical reorganization of the bacterial community.

### Impact of symbionts on the functional profiles of the *C. elegans* microbiome

We next examined the impact of the different symbionts on the functional profiles of *C. elegans* microbiome. Analysis of functional gene alpha-diversity showed that *C. elegans* microbiome exposed to the non-protective and protective *E. faecalis* presented a significantly decreased functional genes richness compared to the control or *P. mendocina* groups (pairwise Kruskal–Wallis, *p* < 0.05, Supplementary Fig. S3A). Significant differences were found in the Shannon entropy of the functional genes of the protective *E. faecalis* group compared to the ancestral, non-protective *E. faecalis* and *P. mendocina* groups (pairwise Kruskal–Wallis, *p* < 0.05, Supplementary Fig. S3B). However, differences in functional genes evenness were only found between the non-protective *E. faecalis* and *P. mendocina* groups (pairwise Kruskal–Wallis, *p* < 0.05, Supplementary Fig. S3C). The comparison of the functional profiles (at the functional EC genes level) using PERMANOVA test on Bray–Curtis dissimilarity index showed that the microbiome of *C. elegans*, when exposed to different symbionts, exhibited significant differences in predicted gene abundance among all the experimental groups (PERMANOVA, *p* < 0.05, data no showed). Similarly, dispersion analysis of the Bray–Curtis dissimilarity index showed significant differences between the samples of the experimental groups (ANOVA, *p* < 0.05, Supplementary Fig. S3D).

**Figure 7:**
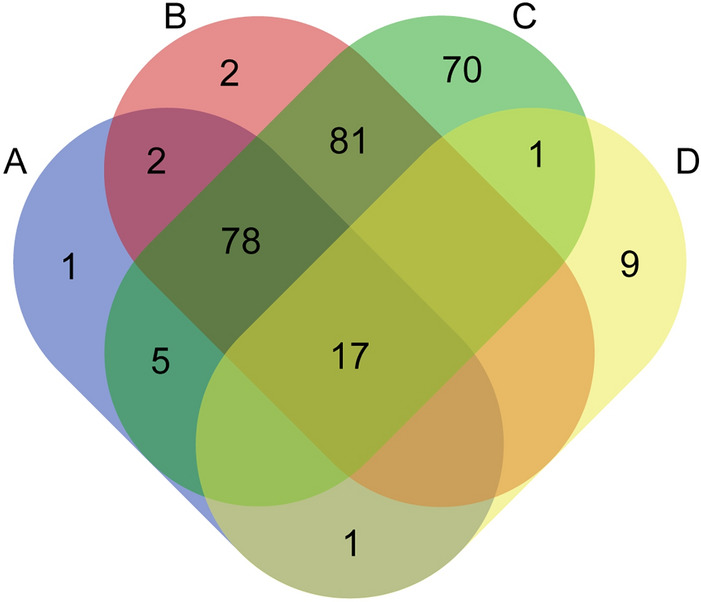
Comparison of unique and share pathways across the different experimental groups. Venn diagram showing the common and unique predicted bacterial pathways, with significant abundance changes, found in the microbiome of C. elegans exposed to the ancestral *E. faecalis* (**A**), non-protective *E. faecalis* (**B**), protective *E. faecalis* (**C**) and *P. mendocina* (**D**) compared to the control group *E. coli* OP50 (Welch’s t-test, *p* < 0.05).

Further analysis of the identity of the different predicted pathways revealed that 92% (i.e., 384/416) of the pathways were shared by all the groups, only one to two pathways were found exclusively in the protective *E. faecalis*, *P. mendocina* and the control group and non-unique pathway were found in the ancestral or non-protective *E. faecalis* groups (Supplementary Fig. S4A). Differential abundance analysis showed that several predicted pathways (Supplementary Fig. S4B) and enzymes (Supplementary Fig. S5) changed significantly among the experimental groups (Kruskal–Wallis, *p* < 0.05). Pairwise comparisons of the pathways between the control (*E. coli* OP50) and the other groups revealed those with differential abundance (Supplementary Table S2). Among the pathways with differential abundance, the protective *E. faecalis* group presented the highest number of unique pathways (i.e., 70), while the other experimental groups presented less than 10 unique pathways with significant differential abundance (Fig. 7). These results suggest that *C. elegans* microbiome exposed to the protective *E. faecalis* symbiont undergoes extensive functional changes and these changes are different from the other experimental groups.

To study the functional departures of the evolved symbionts from the ancestral *E. faecalis*, we performed pairwise comparisons of the predicted pathways. Differential analysis showed no significant differences in pathway abundance between the non-protective and ancestral *E. faecalis*. However, three pathways were found with significant abundance differences between the protective and the ancestral *E. faecalis* (Supplementary Fig. S6, Welch’s t-test, *p* < *0.05*). The abundance of two pathways, l-glutamate and L-glutamine biosynthesis (PWY-5505) and thiazole component of thiamine diphosphate biosynthesis II (PWY-6891) increased significantly while the abundance of the superpathway of thiamine diphosphate biosynthesis II (PWY-6895) decreased in the protective *E. faecalis* group compared to the ancestral *E. faecalis* group (Supplementary Fig. S6).

**Figure 8:**
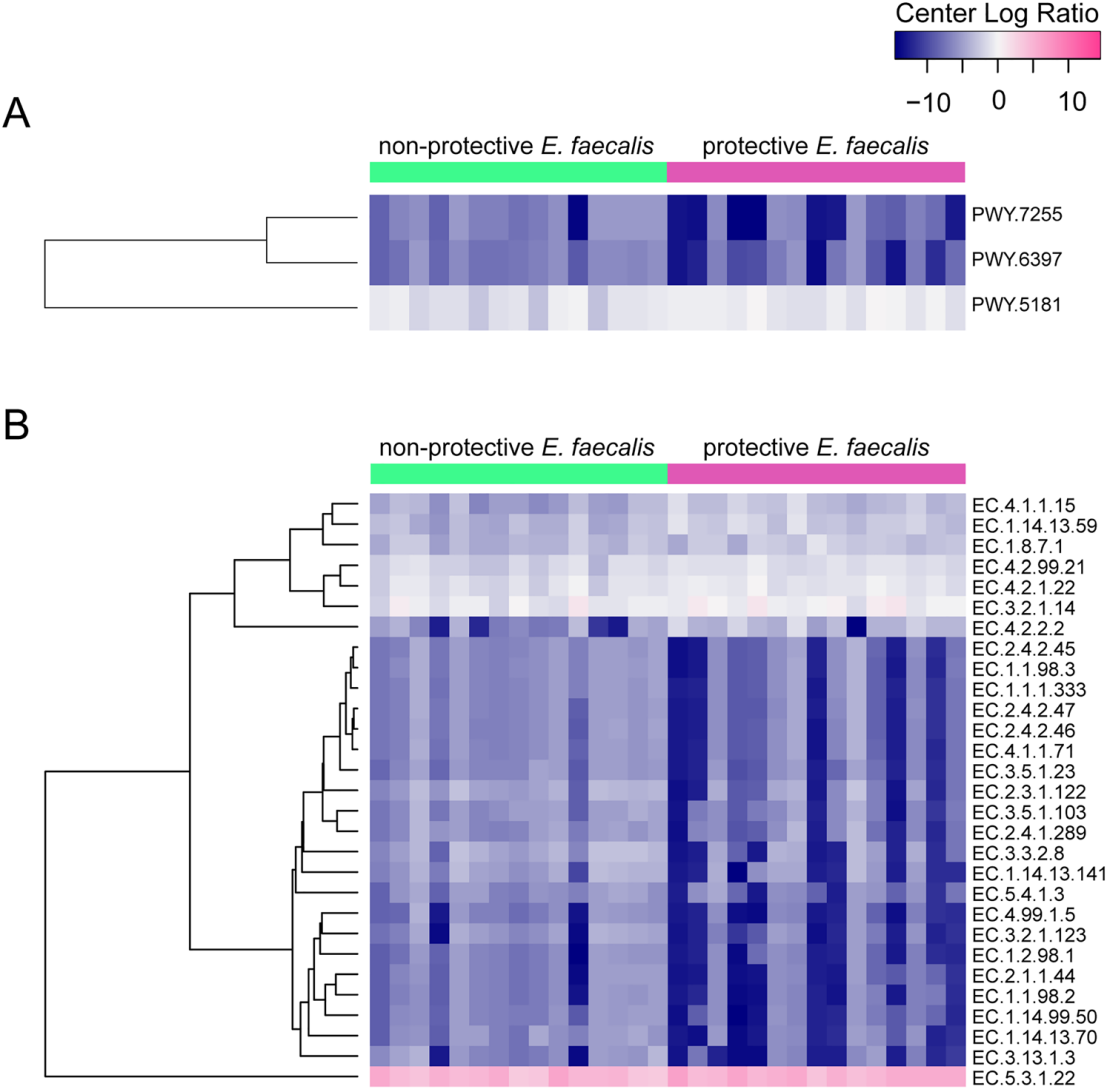
Predicted enzymes and pathways with differential abundance in *C. elegans* exposed to highly protective and less protective *E. faecalis*. Heatmap showing the pathways (**A**) and enzymes (**B**) with significant differences in relative abundance (expressed as clr) between the non-protective and protective *E. faecalis* conditions (Welch’s t-test, *p* < 0.05). PWY-7255: ergothioneine biosynthesis I, PWY-6397: mycolyl-arabinogalactan-peptidoglycan complex biosynthesis, PWY-5181: toluene degradation III (aerobic) (via p-cresol).

We next focused on the comparison between the protective and non-protective *E. faecalis* groups since we were interested in potential mechanisms underpinning the protective role of *E. faecalis*. Differential analysis showed that the abundance of three predicted pathways (Fig. 8A) and 29 enzymes (Fig. 8B) changed significantly between the protective and non-protective *E. faecalis* exposure groups (Welch’s t-test, *p* < 0.05). The abundance of two of these pathways (i.e., ergothioneine (EGT) biosynthesis I (PWY-7255), and mycolyl-arabinogalactan-peptidoglycan complex biosynthesis (PWY-6397)) decreased while the third pathway (i.e., toluene degradation III (PWY-5181)) increased significantly in the protective *E. faecalis* condition compared to non-protective *E. faecalis* group. Accordingly, the abundance of enzymes involved in the ergothioneine biosynthesis pathway such as L-histidine *N*^*α*^-methyltransferase (EC 2.1.1.44) and γ-glutamyl hercynylcysteine S-oxide synthase (EC 1.14.99.50) were significantly lower in the protective *E. faecalis* group compared to the non-protective *E. faecalis* group (Supplementary Fig. S7A). The abundance of the enzyme genes N-acetylglucosaminyl-diphospho-decaprenol L-rhamnosyltransferase (EC 2.4.1.289), galactan 5-O-arabinofuranosyltransferase (EC 2.4.2.46), decaprenyl-phosphate phosphoribosyltransferase (EC 2.4.2.45), decaprenylphospho-β-D-ribofuranose 2-dehydrogenase (EC 1.1.98.3), decaprenylphospho-β-D-erythro-pentofuranosid-2-ulose 2-reductase (EC 1.1.1.333) and arabinofuranan 3-O-arabinosyltransferase (EC 2.4.2.47) involved in mycolyl-arabinogalactan-peptidoglycan complex biosynthesis were significantly lower in the protective symbiont group compared to the non-protective group (Supplementary Fig. S7B). These results highlight the impact of symbiont exposure based on phenotype on microbiome functional profiles.

## Discussion

The disease suppressive benefits of protective microbes to their animal hosts are known^45^. We hypothesized that protective microbes may drive the re-structuring of the host microbial community due to complex microbe-host or microbe-microbe interactions, despite the minimal effects to the composition and diversity of the host microbiota^10^. Here, using available published data^10^, we used a network approach to assess the impact of a protective symbiont on the structure of *C. elegans* microbiota. Although Dahan et al.^10^ previously showed these symbionts had minimal impact on within-host microbiota diversity, our deep-dive into the data showed changes in community assemblages, with alterations in microbial interactions patterns and inferred functional traits. Notably, microbial co-occurrence networks can be used to identify key members of microbial communities referred as keystone taxa^11,23^, and we found that the greatest number of keystone taxa was identified in highly protected hosts, including the genus *Enterococcus* that became a keystone taxon only in the protective *E. faecalis* network. The keystone taxa found in the protective *E. faecalis* network may contribute to the microbial community resiliency to disturbance, and specifically defence against pathogens. Specific keystone bacteria have been found associated with human gut microbiome recovery after another stressor, antibiotic exposure^46^. Moreover, four keystone bacteria were found to maintain the functional diversity of the *Ixodes scapularis* microbiome under intense heat^47^. Whilst the results obtained here may depend on the priority placement of the protective symbionts within the host, earlier establishment of *E. faecalis* is not required for host-protection against pathogens in this system^5,48^. That said, priority effects may still give the symbiont a competitive advantage over some newly arriving commensals.

Although some attributes of the networks differed marginally between the protective *E. faecalis* and the other groups, such as the diameter (a measure of its cohesiveness) or the average degree, other parameters changed noticeably. Microbial networks of highly protected hosts had higher number of nodes and edges suggesting more microbial species interactions and higher complexity in host microbiota. Zheng et al. similarly found that the networks of disease-suppressive soil and healthy root of tobacco plants presented a higher number of nodes and edges and thus, higher network complexity compared to disease-conducive soil and infected roots. Another study showed that reduced microbial network complexity due to agricultural intensification was associated with decreased abundance of keystone taxa in roots^49^. The high complexity network may have been crucial for the suppression of soil-borne pathogens in that study^12^ since complex networks with interacting bacteria can enhance metabolites and information exchange. These additional interactions can make the complex network more efficient^20^.

The network modularity was higher in the evolved symbiont treatments compared to that of the ancestral symbiont. Modularity, which measures the strength of division of a network into modules^21^, can indicate network stability against disturbances such as antibiotic stress^50^ or climate extremes^47,51^. Higher complexity and modularity in the protective network could be associated with higher network robustness. To test this hypothesis, robustness was measured here as the capacity of the network to tolerate sequential removal of nodes without significant loss of network connectivity^52^. Our tolerance test showed that the microbiota community difference induced by protective *E. faecalis* colonisation, compared to that of the control (i.e., *E. coli* OP50), did not alter robustness. Lower robustness and higher complexity in the protective network seem counterintuitive. However, theory predicts that highly structured complexity has the risk of rare but potentially catastrophic cascading failure events initiated by small perturbations^53^. For example, the co-occurrence network of *I. scapularis* microbiota exposed to disturbance from anti-tick antibodies was highly complex with increases in the numbers of nodes and edges, but was less robust to taxa loss compared to undisturbed microbiota^54^. Conversely, while disturbance of the tick microbiota by infection with *Anaplasma phagocytophilum*, a tick-borne bacterium that protects hosts against cold stress^55^, increased network complexity, the robustness did not change, compared with uninfected ticks^54^. While establishing the interplay between network parameters and robustness is not straightforward^56^, our results and others^54^ suggest that protective symbionts can alter global network parameters with reduced impact on network robustness.

We explored the local co-occurrence of the reference taxa within nematodes (i.e., *Enterococcus*, *Pseudomonas* and *Escherichia-Shigella*) across different symbiont groups. We revealed higher local connectivity and stable ecological associations of *Enterococcus* in microbial co-occurrence networks with experimentally-evolved symbionts. This suggests that evolved symbionts form a specific community with other bacteria in the microbiota. Microbial assemblages, phylogenetically defined groups of microbes that co-occur in space and time^57^, can face significant barriers to assembly^58^. Some of these barriers include strong and positive species interactions suggesting that even stable communities may be unable to assemble^58^. Ecological network theory predicts that ecological dependencies between members of the microbiota make assembly predictable, but dependencies may also create barriers to assembly, as interdependent species can fail to establish when each relies on the other to colonize^58^. What explains the commonalities in the local assembly of microbial communities in the presence of evolved *E. faecalis* strains, regardless of the phenotype and/or impact for the host (non-protective *vs*. protective)? We consider two factors. Firstly, core microbe-microbe interactions in evolved *E. faecalis* (Fig. 6C) have a reduced number of positive (i.e., 2/8), and a majority of negative (i.e., 6/8) interactions, which favor community assembly, as cooperating networks of microbes can be efficient, but often unstable^58,59^. In diverse communities, co-occurring species with negative interactions keep one another in check, decreasing the probability of each other extinction and allowing for a stable community^58^. Secondly, modelling and empirical data shows that hosts can overcome barriers to assembly via mechanisms that include providing feeding^58^, immunity^59^, and differential adhesion^60^ to colonizers. Thus, both microbe-microbe and host-microbe interactions are important for microbiome assembly. Whether these two mechanisms act simultaneously or individually to keep the local interactions of evolved *E. faecalis* remains to be explored.

Comparison of the inferred functional genes of *C. elegans* microbiome across symbiont treatments reveals extensive disparity in functional profile. We suggest that, compared to the non-protective *E. faecalis,* the protective symbiont restructures the functional profiles of the microbiome. Such comparisons revealed differential abundance of metabolic pathways exclusively associated with the protective phenotype, not in vivo symbiont evolution alone. Notably, the abundance of one of these pathways, EGT biosynthesis I, decreased in the protective symbiont microbiome. EGT is a trimethylated and sulphurated histidine derivative that exhibits antioxidant properties^61^. Impairment of the EGT synthesis in *Aspergillus fumigatus* leads to a significantly reduced resistance to oxidative stress induced by elevated hydrogen peroxide^61^. Given that the protective role of *E. faecalis* in *C. elegans* against virulent *S. aureus* was mediated by superoxide production^19^, we hypothesize that the decrease in the antioxidant properties of the bacterial community might further enhance the oxidative stress in the nematode gut. This stress might have the effect of creating an environment even more incompatible with pathogen infection^19,62,63^. However, pathway prediction has the limitation of not allowing us to account for strain-specific or species-specific effects. Bacterial metagenome sequencing and/or examination of the metabolite dynamics of *C. elegans* microbiota during protective *E. faecalis* colonization may be informative for testing our hypothesis.

Beyond the general description of microbiota composition and diversity descriptors used in Dahan et al.^10^, we studied correlations between taxa, community structure and functionality of the microbiome across symbiont treatments. Community level analysis can grasp microbial functioning more accurately than compositional analysis alone^21,64^. Ecosystems are shaped by the composition of the community, but also on the type and strength of associations among co-existing members^64–66^. Network-based approaches have proven helpful in deciphering complex microbial interaction patterns^21,64,65^. In addition, as the community response to disturbance often involves changes in microbial interactions without affecting composition or microbial diversity, co-occurrence networks become an essential tool to compare microbial communities under different conditions^51,66^. For example, co-occurrence networks reveal more complexity than community composition in the resistance and resilience of plant microbial communities to drought stress^66^, and using a network approach revealed differential patterns of bacterial interactions in goats infected with the parasite *Cryptosporidium parvum*^67^.

## Conclusions

We found that a rapidly evolved and novel protective symbiont engages in additional interactions with other colonizing micro-organisms to become a keystone taxon. The symbiont had been experimentally evolved in the absence of natural host microbiota, yet it restructured the microbial community. Experimental evolution of protective microbes could be used for microbiome engineering approaches^68,69^ in which microbes are ‘evolutionarily-trained’ to induce desired protective phenotypes amidst the microbiota. Such pathogen colonization resistance traits may also modify microbiota structure and restore ecological balance. Results here suggest that the mechanism of protection may matter, as protection via direct interactions with pathogens (i.e., protective *E. faecalis* vs. *S. aureus*), rather than via host-mediated immunity (*P. mendocina* vs. *P. aeruginosa*) resulted in increased keystoneness for the protective symbiont in the *C. elegans* microbiota. Furthermore, the hierarchical re-organization of the microbial communities may enhance the protective phenotype of *E. faecalis* via functional changes of *C. elegans* microbiota. Application of network biology will enhance our understanding of microbiota responses to protective microbial symbionts. Knowing the extent of cascading effects on microbiomes may prove helpful in determining the side-effects and effectiveness of protective symbionts, which may guide interventions to improve human and animal health.

## Supplementary Information

Supplementary Figure S1.

Supplementary Figure S2.

Supplementary Figure S2.

Supplementary Figure S3.

Supplementary Figure S4.

Supplementary Figure S5.

Supplementary Figure S6.

Supplementary Figure S7.

Supplementary Table S1.

Supplementary Table S2.

## Data Availability

All material relevant to this publication are available in the manuscript. Data used to generate the figures is available in the repository Harvard Dataverse, https://doi.org/10.7910/DVN/S3SVP3.

## References

[1] Samuel BS, Rowedder H, Braendle C, Félix MA, Ruvkun G (2016). *Caenorhabditis elegans* responses to bacteria from its natural habitats. Proc. Natl. Acad. Sci. USA.

[2] Oliver KM, Smith AH, Russell JA (2014). Defensive symbiosis in the real world: Advancing ecological studies of heritable, protective bacteria in aphids and beyond. Funct. Ecol..

[3] King KC (2019). Defensive symbionts. Curr. Biol..

[4] Foster KR, Schluter J, Coyte KZ, Rakoff-Nahoum S (2017). The evolution of the host microbiome as an ecosystem on a leash. Nature.

[5] Ford SA, Kao D, Williams D, King KC (2016). Microbe-mediated host defence drives the evolution of reduced pathogen virulence. Nat. Commun..

[6] Litvak Y (2019). Commensal Enterobacteriaceae protect against *Salmonella* colonization through oxygen competition. Cell Host Microbe.

[7] Pimentel AC, Cesar CS, Martins M, Cogni R (2021). The antiviral effects of the symbiont bacteria *Wolbachia* in insects. Front. Immunol..

[8] Becker MH, Brucker RM, Schwantes CR, Harris RN, Minbiole KPC (2009). The bacterially produced metabolite violacein is associated with survival of amphibians infected with a lethal fungus. Appl. Environ. Microbiol..

[9] Bates KA, Bolton JS, King KC (2021). A globally ubiquitous symbiont can drive experimental host evolution. Mol. Ecol..

[10] Dahan D, Preston GM, Sealey J, King KC (2020). Impacts of a novel defensive symbiosis on the nematode host microbiome. BMC Microbiol..

[11] Banerjee S, Schlaeppi K, van der Heijden MGA (2018). Keystone taxa as drivers of microbiome structure and functioning. Nat. Rev. Microbiol..

[12] Zheng Y (2021). Exploring biocontrol agents from microbial keystone taxa associated to suppressive soil: A new attempt for a biocontrol strategy. Front. Plant Sci..

[13] Tudela H, Claus SP, Saleh M (2021). Next generation microbiome research: Identification of keystone species in the metabolic regulation of host-gut microbiota interplay. Front. Cell Dev. Biol..

[14] Mateos-Hernández L (2020). Anti-tick microbiota vaccine impacts *Ixodes ricinus* performance during feeding. Vaccine.

[15] Mateos-Hernández L (2021). Anti-microbiota vaccines modulate the tick microbiome in a taxon-specific manner. Front. Immunol..

[16] Dirksen P (2016). The native microbiome of the nematode *Caenorhabditis elegans*: Gateway to a new host-microbiome model. BMC Biol..

[17] Berg M (2016). Assembly of the *Caenorhabditis elegans* gut microbiota from diverse soil microbial environments. ISME J..

[18] Zhang F (2017). *Caenorhabditis elegans* as a model for microbiome research. Front. Microbiol..

[19] King KC (2016). Rapid evolution of microbe-mediated protection against pathogens in a worm host. ISME J..

[20] Faust K, Raes J (2012). Microbial interactions: From networks to models. Nat. Rev. Microbiol..

[21] Layeghifard M, Hwang DM, Guttman DS (2017). Disentangling interactions in the microbiome: A network perspective. Trends Microbiol..

[22] Röttjers L, Faust K (2018). From hairballs to hypotheses–biological insights from microbial networks. FEMS Microbiol. Rev..

[23] Agler MT (2016). Microbial hub taxa link host and abiotic factors to plant microbiome variation. PLoS Biol..

[24] Douglas GM (2020). PICRUSt2 for prediction of metagenome functions. Nat. Biotechnol..

[25] Hou Y (2021). Hierarchical microbial functions prediction by graph aggregated embedding. Front. Genet..

[26] Montalvo-Katz S, Huang H, Appel MD, Berg M, Shapira M (2013). Association with soil bacteria enhances p38-dependent infection resistance in *Caenorhabditis elegans*. Infect. Immun..

[27] Callahan BJ (2016). DADA2: High-resolution sample inference from Illumina amplicon data. Nat. Methods.

[28] Bolyen E (2019). Reproducible, interactive, scalable and extensible microbiome data science using QIIME 2. Nat. Biotechnol..

[29] Bokulich NA (2018). Optimizing taxonomic classification of marker-gene amplicon sequences with QIIME 2’s q2-feature-classifier plugin. Microbiome.

[30] Yarza P (2014). Uniting the classification of cultured and uncultured bacteria and archaea using 16S rRNA gene sequences. Nat. Rev. Microbiol..

[31] Friedman J, Alm EJ (2012). Inferring correlation networks from genomic survey data. PLoS Comput. Biol..

[32] RStudio Team (2020). RStudio: Integrated Development for R.

[33] Bastian, M., Heymann, S. & Jacomy, M. Gephi: An open-source software for exploring and manipulating networks. *Third International AAAI Conference on Weblogs and Social Media* (2009).

[34] Lhomme, S. *NetSwan: Network Strengths and Weaknesses Analysis. R Pack Version* (2015).

[35] Peschel S, Müller CL, von Mutius E, Boulesteix AL, Depner M (2021). NetCoMi: Network construction and comparison for microbiome data in R. Brief Bioinform..

[36] Kanehisa M (2000). Goto, S, KEGG: Kyoto Encyclopedia of Genes and Genomes. Nucleic Acids Res..

[37] Tatusov RL, Galperin MY, Natale DA, Koonin EV (2000). The COG database: A tool for genome-scale analysis of protein functions and evolution. Nucleic Acids Res..

[38] Caspi R (2018). The MetaCyc database of metabolic pathways and enzymes. Nucleic Acids Res..

[39] Fernandes AD (2014). Unifying the analysis of high-throughput sequencing datasets: Characterizing RNA-seq, 16S rRNA gene sequencing and selective growth experiments by compositional data analysis. Microbiome.

[40] Lin H, Peddada SD (2020). Analysis of microbial compositions: A review of normalization and differential abundance analysis. npj Biofilms Microbiomes.

[41] Ploner, A. *Heatplus: Heatmaps with Row and/or Column Covariates and Colored Clusters. R package version 3.2*. (2021).

[42] Shannon, C. E. A mathematical theory of communication. *Bell Syst. Tech. J.* **27**, 379–423, 623–656 (1948).

[43] Pielou EC (1966). The measurement of diversity in different types of biological collections. J. Theor. Biol..

[44] Fisher RA, Corbet AS, Williams CB (1943). The relation between the number of species and the number of individuals in a random sample of an animal population. J. Anim. Ecol..

[45] Ford SA, King KC (2016). Harnessing the power of defensive microbes: Evolutionary implications in nature and disease control. PLoS Pathog..

[46] Gibbons SM (2020). Keystone taxa indispensable for microbiome recovery. Nat. Microbiol..

[47] Wu-Chuang A (2021). Thermostable keystone bacteria maintain the functional diversity of the *Ixodes scapularis* microbiome under heat stress. Microb. Ecol..

[48] Ford SA, King KC (2021). In vivo microbial coevolution favors host protection and plastic downregulation of immunity. Mol. Biol. Evol..

[49] Banerjee S (2019). Agricultural intensification reduces microbial network complexity and the abundance of keystone taxa in roots. ISME J..

[50] Gao Q (2021). The microbial network property as a bio-indicator of antibiotic transmission in the environment. Sci. Total Environ..

[51] de Vries FT (2018). Soil bacterial networks are less stable under drought than fungal networks. Nat. Commun..

[52] de Morais, U. L. A look at the way we look at complex networks’ robustness and resilience. https://arxiv.org/ftp/arxiv/papers/1909/1909.06448.pdf (2017).

[53] Carlson JM, Doyle J (2002). Complexity and robustness. Proc. Natl. Acad. Sci. USA.

[54] Estrada-Peña A, Cabezas-Cruz A, Obregón D (2020). Resistance of tick gut microbiome to anti-tick vaccines, pathogen infection and antimicrobial peptides. Pathogens.

[55] Neelakanta G, Sultana H, Fish D, Anderson JF, Fikrig E (2010). Anaplasma phagocytophilum induces *Ixodes scapularis* ticks to express an antifreeze glycoprotein gene that enhances their survival in the cold. J. Clin. Investig..

[56] Dey AK, Gel YR, Poor HV (2019). What network motifs tell us about resilience and reliability of complex networks. Proc. Natl. Acad. Sci. USA.

[57] Nemergut DR (2013). Patterns and processes of microbial community assembly. Microbiol. Mol..

[58] Coyte KZ, Rao C, Rakoff-Nahoum S, Foster KR (2021). Ecological rules for the assembly of microbiome communities. PLoS Biol..

[59] Coyte KZ, Schluter J, Foster KR (2015). The ecology of the microbiome: Networks, competition, and stability. Science.

[60] McLoughlin K, Schluter J, Rakoff-Nahoum S, Smith AL, Foster KR (2016). Host selection of microbiota via differential adhesion. Cell Host Microbe.

[61] Sheridan KJ (2016). Ergothioneine biosynthesis and functionality in the opportunistic fungal pathogen, *Aspergillus fumigatus*. Sci. Rep..

[62] Rothfork JM (2004). Inactivation of a bacterial virulence pheromone by phagocyte-derived oxidants: New role for the NADPH oxidase in host defense. Proc. Natl. Acad. Sci. USA.

[63] Gaupp R, Ledala N, Somerville GA (2012). Staphylococcal response to oxidative stress. Front. Cell. Infect. Microbiol. Microbiol..

[64] Matchado MS (2021). Network analysis methods for studying microbial communities: A mini review. Comput. Struct. Biotechnol. J..

[65] Jiang D (2019). Microbiome multi-omics network analysis: Statistical considerations, limitations, and opportunities. Front. Genet..

[66] Gao C (2022). Co-occurrence networks reveal more complexity than community composition in resistance and resilience of microbial communities. Nat. Commun..

[67] Mammeri M (2020). *Cryptosporidium parvum* infection depletes butyrate producer bacteria in goat kid microbiome. Front. Microbiol..

[68] Foo, J. L., Ling, H., Lee, Y. S. & Chang, M. W. Microbiome engineering: Current applications and its future. *Biotechnol. J.* **12**, 1600099 (2017). 10.1002/biot.201600099 28133942

[69] Inda, M. E., Broset, E., Lu, T. K. & de la Fuente-Nunez, C. Emerging frontiers in microbiome engineering. *Trends Immunol.* **40**, 952–973 (2019). 10.1016/j.it.2019.08.007 31601521

