# Supplementary figures and images for: Rapid evolution of a novel protective symbiont into keystone taxon in Caenorhabditis elegans microbiota
Source: Sci Rep. 2022 Aug 18;12:14045. doi: 10.1038/s41598-022-18269-7 (PMC9388637; doi:10.1038/s41598-022-18269-7)

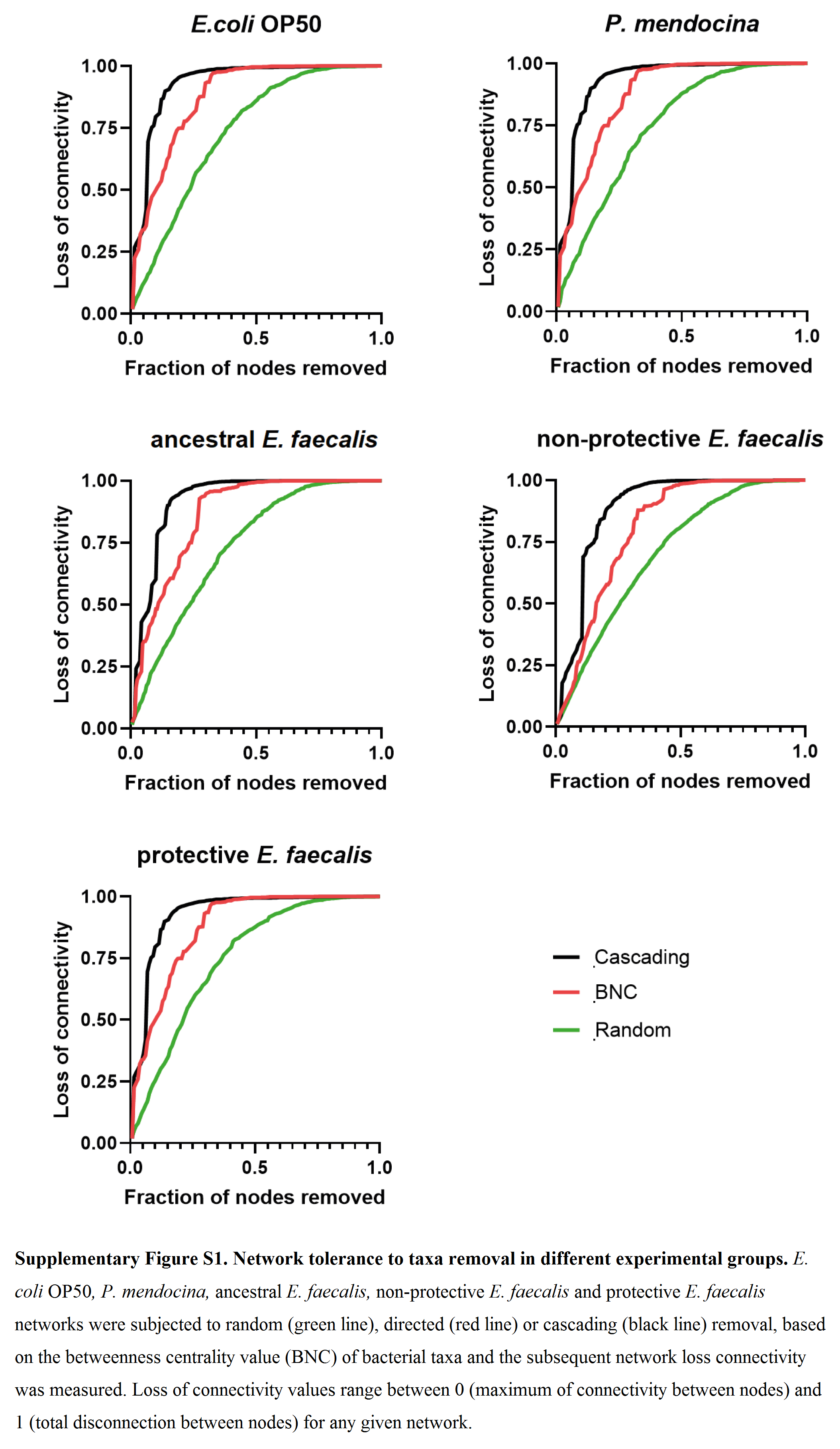

Supplement: Supplementary file 1 — Supplementary Figure S1. [file 41598_2022_18269_MOESM1_ESM.tif]

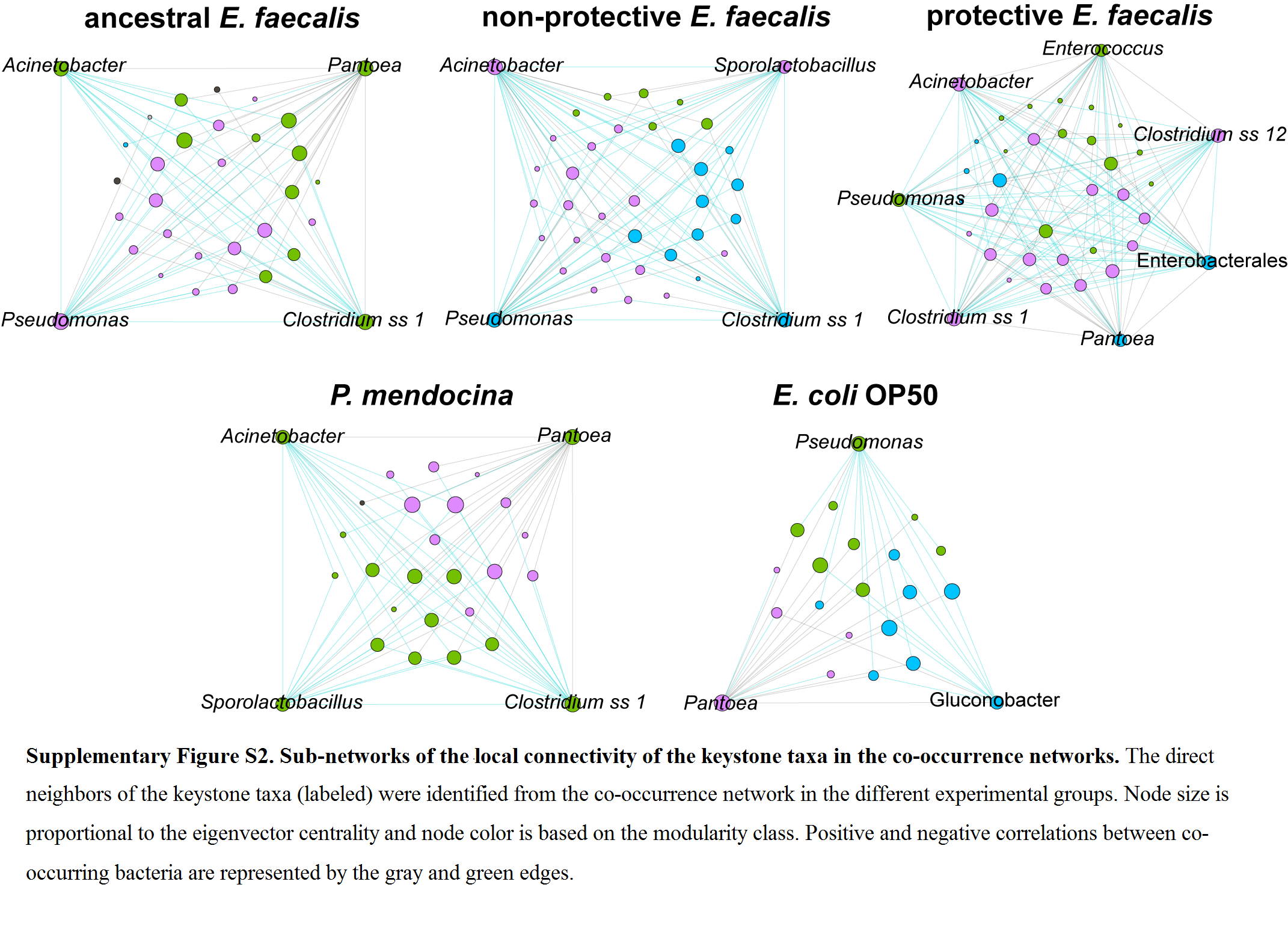

Supplement: Supplementary file 2 — Supplementary Figure S2. [file 41598_2022_18269_MOESM2_ESM.tif]

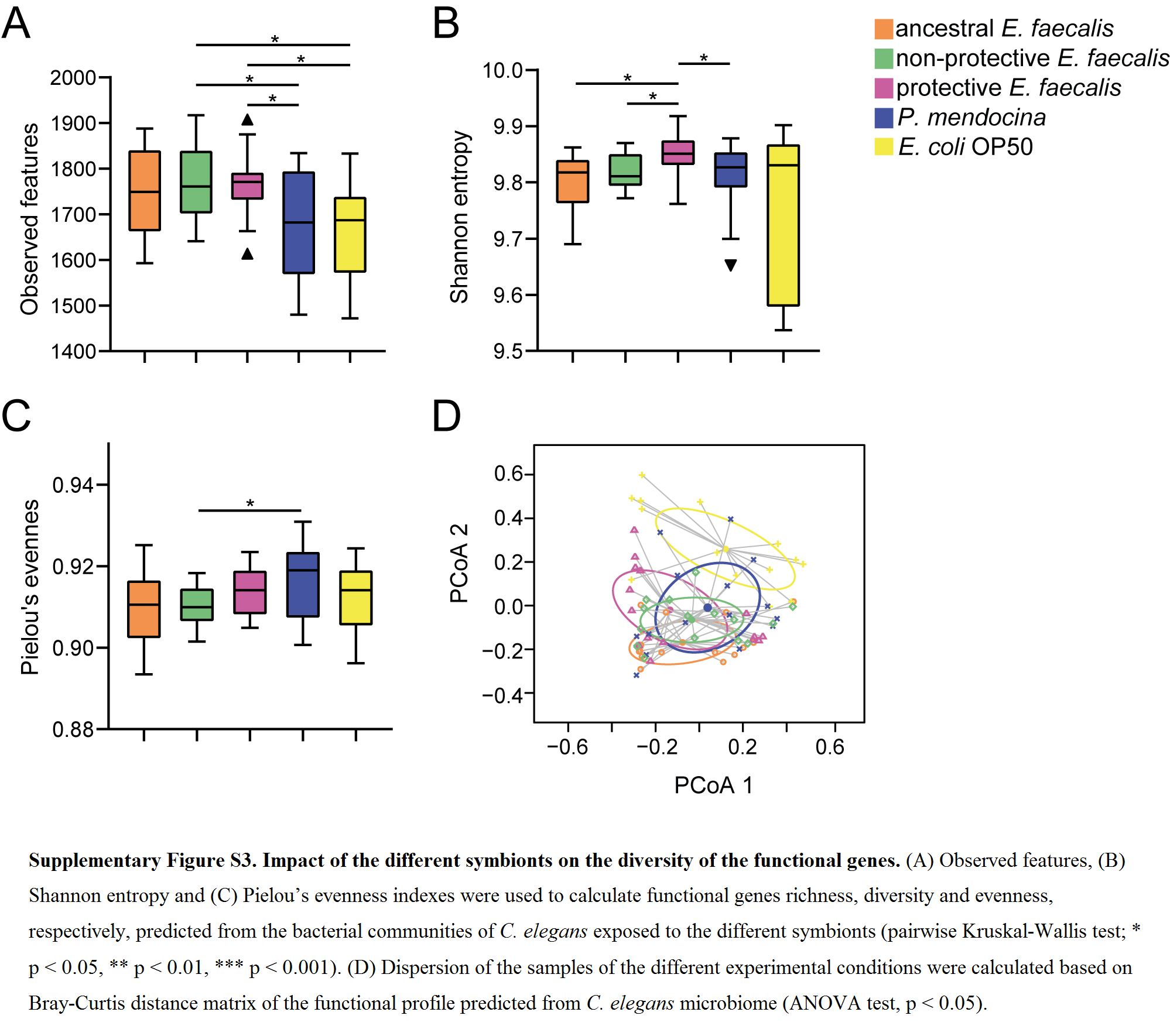

Supplement: Supplementary file 4 — Supplementary Figure S3. [file 41598_2022_18269_MOESM4_ESM.tif]

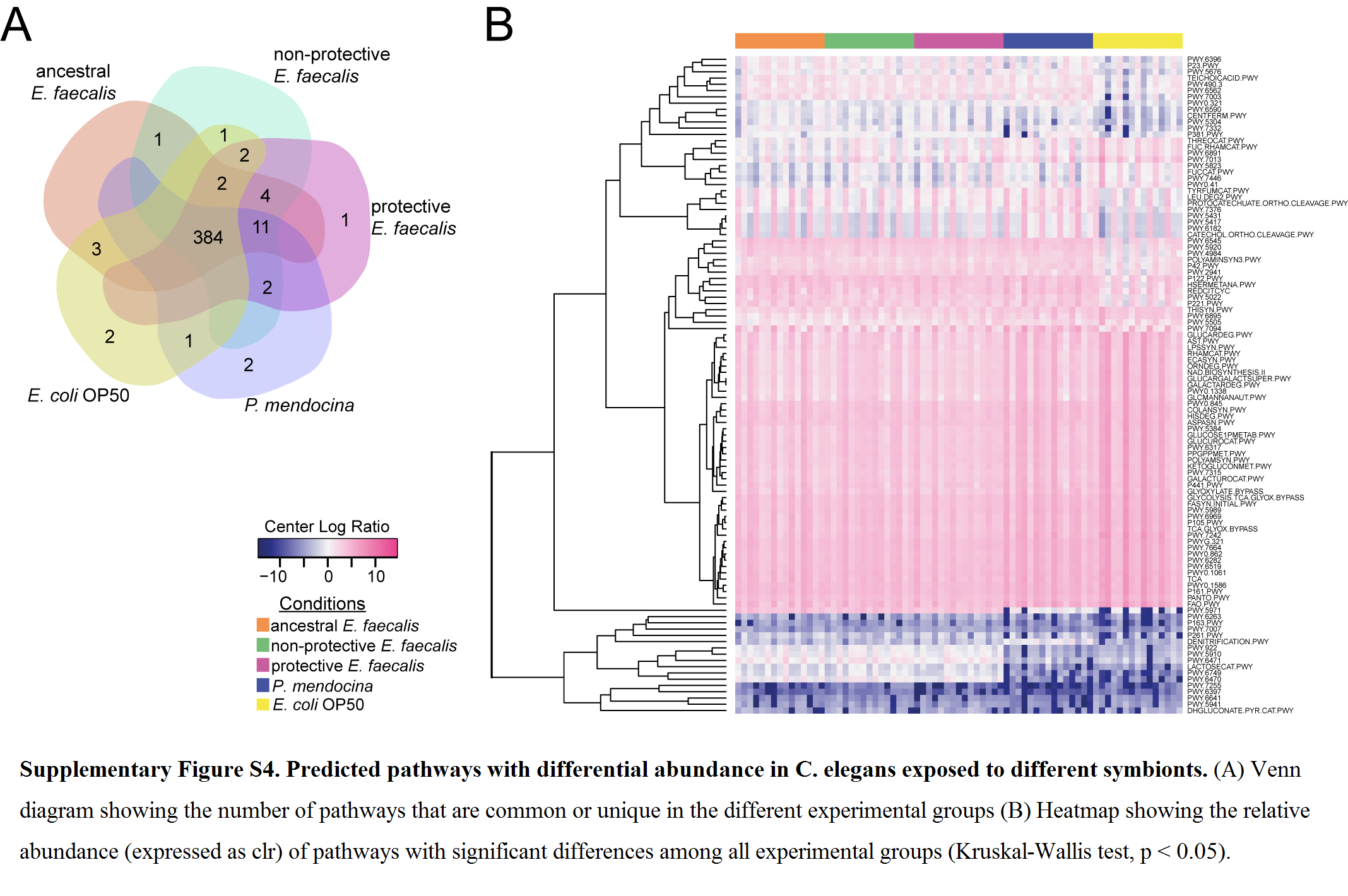

Supplement: Supplementary file 5 — Supplementary Figure S4. [file 41598_2022_18269_MOESM5_ESM.tif]

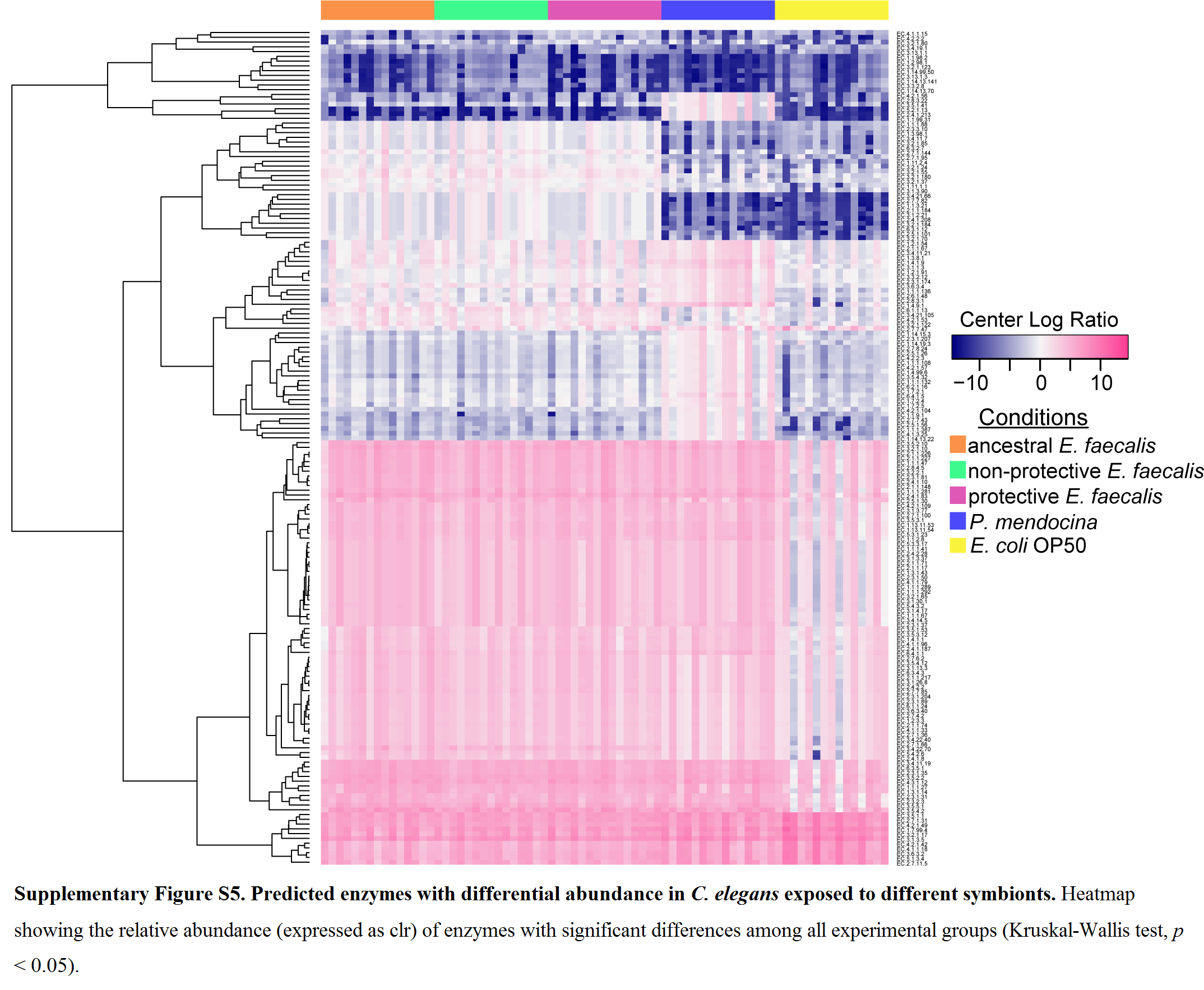

Supplement: Supplementary file 6 — Supplementary Figure S5. [file 41598_2022_18269_MOESM6_ESM.tif]

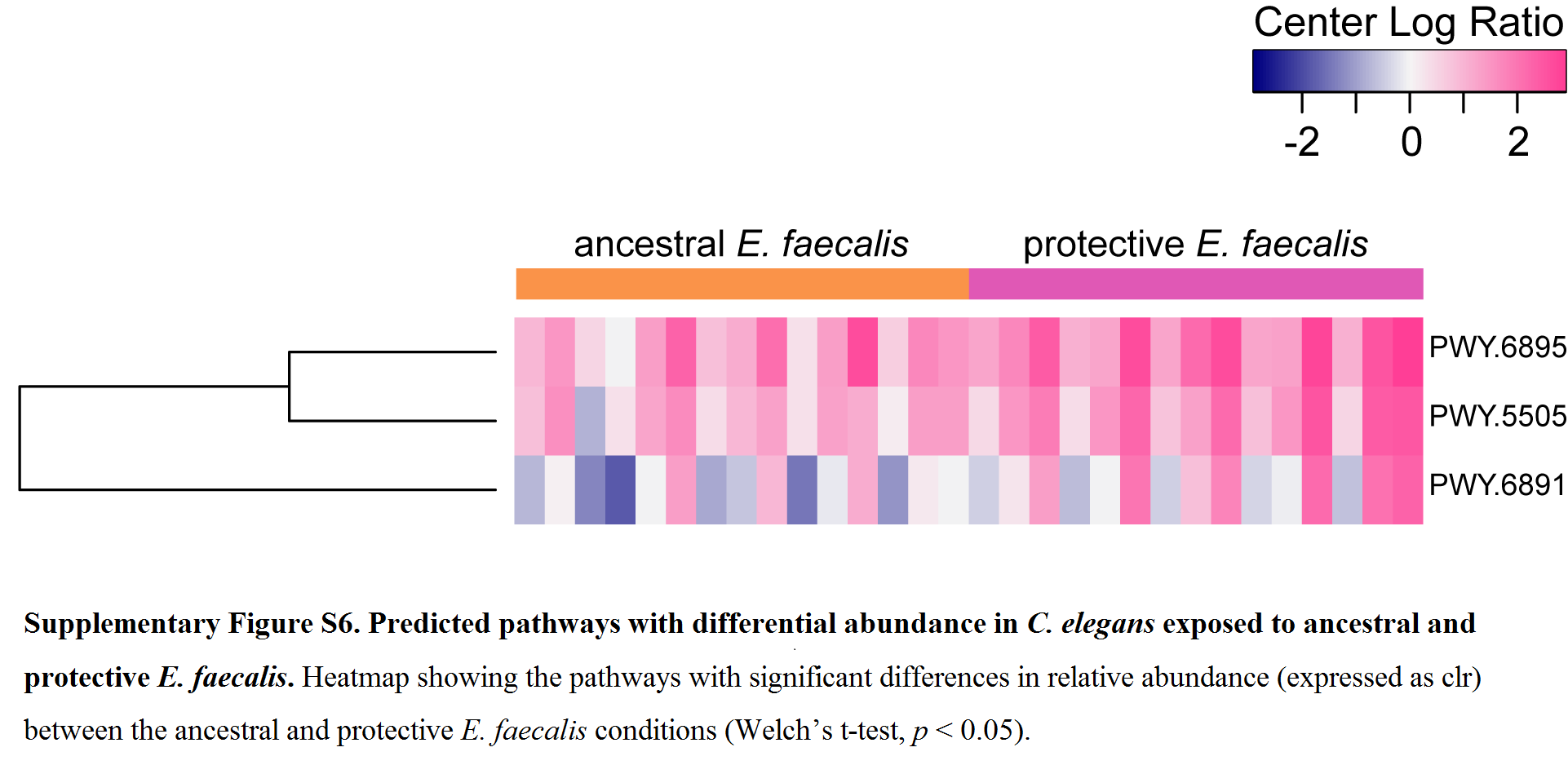

Supplement: Supplementary file 7 — Supplementary Figure S6. [file 41598_2022_18269_MOESM7_ESM.tif]

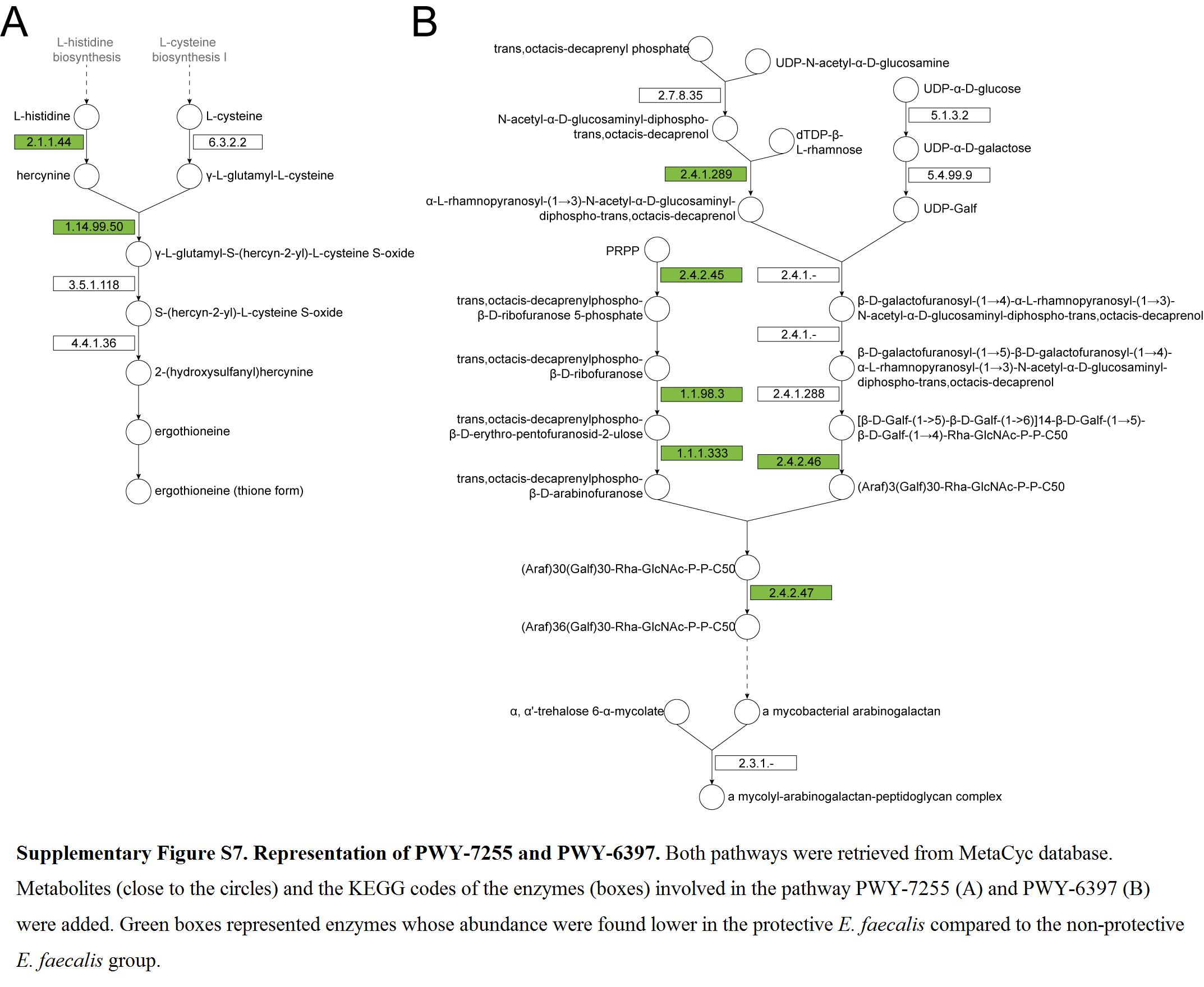

Supplement: Supplementary file 8 — Supplementary Figure S7. [file 41598_2022_18269_MOESM8_ESM.tif]
